# Child Sexual Abuse Material Users on the Darknet: Psychiatric Morbidities Related to Offence Behavior

**DOI:** 10.1177/10790632251347562

**Published:** 2025-05-29

**Authors:** Johanna Lätth, Malin Joleby, Allison McMahan, Timothy J. Luke, Christoffer Rahm

**Affiliations:** 1 27106 Centre for Psychiatry Research, Department of Clinical Neuroscience, Karolinska Institutet, and Stockholm Health Care Services, Region Stockholm, Stockholm, Sweden; 2 Department of Psychology, Gothenburg University, Gothenburg, Sweden

**Keywords:** pedophilia, sexual interests, child sexual abuse material, hypersexuality, zoophilia

## Abstract

Individuals engaging in child sexual abuse often present pedophilic interest, but the other psychiatric morbidities among undetected users of child sexual abuse material (CSAM) are largely unknown. We mapped the psychiatric profile of 160 mainly male and primarily non-convicted anonymous Darknet-recruited adult CSAM users. The participants’ psychiatric morbidities were analyzed descriptively, and correlations between sexual pathologies known as risk factors for committing child sexual abuse (paraphilias, hypersexuality) and CSAM offense behavior (viewing time, content) were examined in a series of exploratory linear regression models. Pedophilic interests, hypersexuality, autism traits, ADHD, and depression were commonly reported. Further, hypersexuality was associated with CSAM viewing time (*r* = .295, *b* = 0.07, *p* = .001), zoophilic interest was associated with CSAM severity (*r* = .195, *b* = 0.46, *p* = .003) and both zoophilic and pedophilic sexual interest were associated with the age of the youngest child in CSAM viewed (*r* = −.218, *b* = −0.56, *p* = .01 and *r* = −.273, *b* = −1.01, *p* < .001). We conclude that CSAM users presented sexual pathologies, some related to their CSAM-use behavior, as well as multiple other mental health needs.

## Introduction

In recent decades, global internet access has greatly changed the opportunity structure for sexual violence against children (Europol, 2020). Online offending and exploitation are rapidly increasing (European Union, 2023), with an alarming 36 million reports of suspected child sexual abuse material (CSAM) to international tip lines in 2023 (NCMEC, 2024). It is estimated that around four percent of the adult male population occasionally uses CSAM for sexual purposes (Seto et al., 2015). In addition, CSAM content is becoming more severe and involves younger children than in the past (Internet Watch Foundation, 2022). Notably, in a survey on the overlay network referred to as “the Darknet”, eight percent of CSAM users reported viewing imagery involving children aged 0–3 years (Insoll et al., 2022).

To address the rising incidence of CSAM-related offenses, mental health interventions for at-risk individuals are recommended as a key strategy (Seto et al., 2024; WHO, 2016) and helplines and specialized outpatient clinics have been established across many Western countries (Adebahr et al., 2021; Beier et al., 2015; Newman et al., 2024). In treatment, prioritized attention should be given to morbidities that have a direct association with offending behaviors, especially those that are potentially treatable risk factors for sexual offending. A thorough understanding of other mental health needs is also required to enable a tailored and holistic treatment approach in terms of content and methods.

Previous studies investigating psychiatric morbidities among CSAM users have been conducted primarily on convicted users and report high levels of pedophilia (Babchishin et al., 2018; Krueger et al., 2009), hypersexuality, depression, and anxiety (Krueger et al., 2009). Abé with colleagues (2021), on the other hand, studied mainly non-convicted men with pedophilic disorder and found neuropsychiatric traits common (autism traits 60%, ADHD traits 38%). Among those, almost one-third reported past week CSAM use (Wittström et al., 2020). To our knowledge, a more detailed description of the psychiatric morbidities of CSAM users has not been made. Furthermore, the extent to which the characteristics of detected CSAM users apply to the likely large (Drury et al., 2020) group of undetected users is vastly unstudied. Providing a broad description of mental health needs among users of CSAM can help identify treatment targets related to their CSAM use behavior as well as their general well-being.

Identifying which morbidities are directly linked to CSAM use presents a significant challenge (Ly et al., 2018). Sexual offenses are multifaceted behaviors driven by multiple motives, varying at the individual and group levels (Robertiello & Terry, 2007). However, factors related to atypical sexuality (i.e., offense-related paraphilic interests and hypersexuality) appear to play a crucial role. Atypical sexuality is one of the identified domains of dynamic (i.e., potentially changeable) risk factors for sexual recidivism (Hanson & Morton-Bourgon, 2005; Seto et al., 2023). In the Motivation-Facilitation Model, these factors are proposed as motivators for the onset of offending (Seto, 2019).

Atypical sexuality appears to be a particularly relevant factor contributing to CSAM-related offenses (e.g. Babchishin et al., 2015). Evidence indicates that convicted CSAM users exhibit higher levels of atypical sexuality compared to individuals convicted of contact child sexual abuse (CSA) (Babchishin et al., 2015, 2018; Klein et al., 2015; Ly et al., 2016). Specifically, CSAM users (with or without a history of contact CSA) report pedophilic interests more frequently than those convicted solely of contact CSA (Babchishin et al., 2015; Ly et al., 2016). Consistent with this finding, a sample of predominantly non-convicted CSAM users reported a pedophilic interest prevalence of 85%, with 60% likely meeting criteria for pedophilic disorder, alongside high levels of hypersexuality (Lätth et al., 2022).

Co-occurrence of multiple paraphilias is common (Abel et al., 1988), and this phenomenon may similarly apply to CSAM users (Elbert et al., 2021). Despite its relevance, the role of paraphilias other than pedophilia in CSAM use remains under-researched. In particular, zoophilia and sexual sadism warrant close examination. Both paraphilias have in previous literature been associated with the most severe forms of child sexual abuse (Canadian Centre for Child Protection, 2018; Reale et al., 2022), and sexual sadism is identified in the Motivation-Facilitation Model (Seto, 2019) as a motivating factor. Investigations into the association of these paraphilias with CSAM content preferences could advance the understanding of CSAM use.

The aim of this study was twofold. First, we aimed to provide a broad description of psychiatric morbidities among CSAM users, including sexual pathologies but also other mental health needs of relevance for treatment content and methods. Based on previous research indicating a high prevalence of neurodevelopmental disorders among males with pedophilic disorder, these conditions were of special interest to us. Second, we aimed to investigate the associations between atypical sexuality and aspects of CSAM offense behaviors. We employed an exploratory analysis approach by using data from a previous trial of anonymous, primarily non-convicted and mainly Darknet-recruited CSAM users (Lätth et al., 2022). Based on the abovementioned previous research in the field, we were interested in exploring whether specific offense-related paraphilic (pedophilic, sexual sadistic, and zoophilic) interests and hypersexuality are associated with aspects of CSAM use behavior. More specifically, we chose to explore the associations between hypersexuality, pedophilic, sexual sadistic, and zoophilic interest and CSAM viewing time, CSAM severity, and the age of the youngest child in CSAM viewed.

## Methods

This study is part of a global participant-blinded randomized placebo-controlled trial conducted online from the ANOVA clinic at the Karolinska University Hospital and the Centre for Psychiatry Research, Karolinska Institutet, Stockholm, Sweden, from April 16, 2019, to December 17, 2021. We used the ISRCTN Registry for pre-registration of the trial (ISRCTN76841676), and the Karolinska Trial Alliance as an external study monitor. The project applied a public- and patient-involvement approach, involving patient and child rights representatives in the planning. Although data from this clinical trial have been analyzed in previous research (Lätth et al., 2022), the analyses conducted in the present study are entirely new and have not been previously reported. We report the study according to the Strengthening of the Reporting of Observational Studies in Epidemiology (STROBE) statement. The research project was approved by the Swedish Ethics Review Appeals Board (no Ö2019-1) on March 18, 2019. The authors assert that all procedures contributing to this work comply with the Helsinki Declaration of 1975, as revised in 2008.

### Participants

This study is based on the same sample as the Lätth et al. (2022) trial (*n* = 160). Eligible participants were at least 18 years old, had viewed CSAM during the past week, had sufficient skills in the English language, and provided informed consent to participate. We excluded individuals who lacked serious intent to participate (*n* = 1) or suffered severe psychiatric illness (*n* = 2), specified as acute suicidal ideation hindering study participation, psychosis, intellectual disability, or severe substance misuse. Participants were anonymous and recruited online, mainly over the Darknet, an encrypted overlay network requiring specific browsers such as The Onion Router (Tor), in a range of chat forums focused on child sexual interest and abuse, but none specifically related to other particular paraphilic interest (Darknet, *n* = 114; through Virtuous Pedophiles available on both the Darknet and clearnet, *n* = 14; elsewhere, *n* = 25; no data, *n* = 7). We posted a link to the study in chats and topic links and answered questions. Participants self-selected by applying through the study web page, where they received written study information and had the opportunity to ask questions. When applying, participants were assigned a random study ID, and could choose whether to register an email address. Research team members conducted assessment interviews, mainly through online text chat but in a few cases by anonymous audio calls on the platform. We assessed eligibility criteria during the interview. All participants provided written informed consent.

About half of the participants were young adults, with participants falling into the following age brackets (%): 18–29 (49); 30–39 (30); 40–49 (15); 50–59 (6), and genders: male 157; non-binary 2; chose not to report 1. A minority reported prior convictions (contact sexual offenses, *n* = 4; non-contact sexual offenses, *n* = 7; non-sexual offenses, *n* = 6). All world regions were represented in the sample; however, the majority of the participants lived in Europe or North America. For more demographics, see Lätth et al. (2022).

The sample size was determined by the power analysis of the preceding trial. Please see a description of the sensitivity analysis for this study in the Statistical Analysis section.

### Materials

The outcomes were (i) time spent viewing CSAM the past week (hours and minutes), (ii) age of the youngest child in CSAM viewed the past week (years), and (iii) severity of CSAM content viewed the past week. We investigated self-reported sexual conditions as potential predictors of the outcomes, including hypersexuality and pedophilic, sexual sadistic, and zoophilic sexual interests. Please note that the term *predictor* refers to the role in the regression models and does not necessarily imply causality.

All outcomes were self-reported using the Sexual Child Molestation Risk Assessment+ (SChiMRA+, part B). This is a timeline follow-back questionnaire that examines offenses and offense-related behaviors over the past seven days (Landgren et al., 2020). SChiMRA+, part B, item one, focuses on behaviors related to watching children for sexual arousal. It also incorporates a self-report version of the Combating Paedophile Information Networks in Europe (COPINE) scale (Quayle, 2008). The COPINE scale measures the severity of CSAM on a scale from 1 to 10, where 1 = indicative, 2 = nudist, 3 = erotic, 4 = posing, 5 = erotic posing, 6 = explicit erotic posing, 7 = explicit sexual activity, 8 = assault, 9 = gross assault, and 10 = sadistic/bestiality. Additionally, participants provide an estimate of the age of the youngest child viewed.

Hypersexuality was measured with the Hypersexual Behavior Inventory-19 (HBI-19) (Reid et al., 2011), a 5-point Likert scale encompassing 19 items divided into three subscales: coping, control, and consequences. The scale ranged from 19 to 95 points, with a cutoff set at 53+ points. HBI-19 has demonstrated strong reliability among males seeking help for hypersexual problems (full scale α = .95; subscales: coping α = .90, control α = .94, consequences, α = .87) (Reid et al., 2011) and acceptable to good reliability in a non-clinical sample (subscales: coping α = .86, control α = .82, consequences, α = .75) (Bőthe et al., 2018). In the present data, HBI-19 had adequate reliability (ω = .76, α = .95).

Paraphilic interests were measured with the Self-assessment of Sexual Interest (SSI) screening for paraphilic disorders (unpublished document developed by Dr Niklas Långström). SSI measures DSM-5 diagnostic criteria for paraphilic disorders. Pedophilic, sexually sadistic, and zoophilic interests were defined as self-reporting *somewhat agreeing* or *completely agreeing* to have those interests. Having probable paraphilic disorders requires additional factors, which are specified in Table 1.

**Table 1.** Psychiatric Characteristics.

Measure	*N*	M (SD)	Median	*N* above cutoff (%)
HBI-19^a^	151	63.5 (17.9)	64	111 (74)
Paraphilia screening^b^				
Pedophilic interest	151	2.44 (0.94)	3	129 (85)
Probable pedophilic disorder^c^	151			91 (60)
Sexual sadism interest	151	1.11 (1.28)	0	58 (38)
Probable sexual sadism disorder^d^	151			12 (8)
Zoophilic interest	151	0.70 (1.13)	0	38 (25)
Probable zoophilic disorder^e^	151			0 (0)
RAADS^f^	154	14.1 (10.4)	13.5	Cutoff 14 + 77(50)
				Cutoff 23 + 33(21)
ASRS^g^	154	11.9 (4.7)	12	57 (37)
MADRS-S^h^	153	16.4 (9.4)	16	Mild 47 (31)
				Moderate 44 (29)
				Severe 7 (5)
AUDIT^i^	154	3.7 (4.9)	2	Risk drinking 22 (14)
				Dependence 7 (5)
DUDIT^j^	154	2.3 (5.7)	0	18 (12)
MINI^k^				
Anti-sociality	156			15 (10)
Depression	156			Current 15 (10)
				Previously 2 (1)
				Recurrent 37 (24)
Generalized anxiety disorder	154			24 (16)
Social anxiety	157			28 (18)
Mania	157			0 (0)
Hypomania	157			0 (0)
Panic disorder	157			11 (7)
PTSD	157			7 (4)
Psychosis	155			1 (0)
Suicidality	157			Low 18 (12)
				Moderate 16 (10)
				High 17 (11)
OCD^l^	156			13 (8)
Anorexia	154			0 (0)
Bulimia	154			0 (0)
Binge eating	153			5 (3)
SChiMRA+				
Viewing (hours/week)	154	5.61 (8.44)	3.21	
Copine	151	7.72 (1.96)	8.50	
Youngest child (y)	151	7.31 (3.34)	7.60	

^a^HBI-19 cutoff 53 or higher out of 93 points.

^b^Screening for paraphilias based on DSM-5 criteria. Having an interest means to *somewhat agree* or *completely agree* to having that interest, for pedophilia “I have had sexually arousing fantasies about, or felt urges to, have sex with someone who is prepubescent (and at least 5 years younger than myself)”, for sexual sadism “I have had sexually arousing fantasies or urges about someone else getting bound, spanked or otherwise getting exposed to pain, humiliation or suffering”, and for zoophilia “I have had sexually arousing fantasies about, or felt urges to, have sex with animals”.

^c^Probably meeting DSM-5 criteria defined as reporting pedophilic interest as described above and reporting *somewhat agree* or *completely agree* to “At some point this lasted for a period of six months or more” and fulfilling study inclusion criteria of past week CSAM use and being 18+ years old.

^d^Probably meeting DSM-5 criteria defined as reporting sadistic sexual interest as described above and reporting *somewhat agree* or *completely agree* to “At some point this lasted for a period of six months or more” and reporting *somewhat agree* or *completely agree* to “I am currently experiencing such a period” and responding *somewhat agree* or *completely agree* to “I have bounded, spanked, beaten or otherwise exposed someone else to pain, humiliation or suffering and became sexually aroused by this” or “The sexual fantasies, urges and behaviors caused/causes me distress or impairment in social occupational or other areas of functioning”, and fulfilling study inclusion criteria of being 18+ years old.

^e^Probably meeting DSM-5 criteria defined as reporting zoophilic interest as described above and reporting *somewhat agree* or *completely agree* to “At some point this lasted for a period of six months or more” and reporting *somewhat agree* or *completely agree* to “I am currently experiencing such a period” and responding *somewhat agree* or *completely agree* to “I have had sex with animals” or “The sexual fantasies, urges and behaviors caused/causes me distress or impairment in social occupational or other areas of functioning”, and fulfilling study inclusion criteria of being 18+ years old.

^f^RAADS cutoffs 14 or higher and 23 or higher out of 42 points.

^g^ASRS cutoff 14 or higher out of 24 points.

^h^MADRS-S mild depression 13–19 points, moderate depression 20–34 points, severe depression 35 points or more out of 54 points.

^i^AUDIT cutoff 8 or more for risk drinking, 15 or more for alcohol dependence out of 40 points (cutoffs for males used).

^j^DUDIT cutoff 6 or more out of 44 points (cutoff for males used).

^k^Based on screening according to DSM-IV criteria.

^l^with a non-pedophilic focus.

Traits of attention-deficit/hyperactivity disorder (ADHD) were measured with the Adult ADHD Self-Report Scale screener (ASRS screener; Kessler et al., 2005), a 5-point Likert scale including 18 items. The scale ranges from 0 to 24 points, with a cutoff at 14+ points. Kessler et al. (2007) reported internal consistency reliability of α = .63–.72. In the present data, ASRS had adequate reliability (ω = .78, α = .86).

Autism traits were measured with the Ritvo Autism and Asperger Diagnostic Scale Screen 14 (RAADS-14) (Eriksson et al., 2013), a 4-point Likert scale encompassing subscales for mentalization deficits, social anxiety, and sensory reactivity. RAADS-14 ranges from 0 to 42 points, with cutoffs at 14+ (presenting a sensitivity of 97% and a specificity of 46–64%) and 23+ points. Eriksson et al. (2013) reported strong internal consistency (α = .90). In the present study, RAADS-14 had adequate reliability (ω = .65, α = .87).

Alcohol abuse was measured with the Alcohol Use Disorder Test (AUDIT; Bergman & Källmén, 2002), a 5-point Likert scale encompassing 10 items. The score ranged from 0 to 40 points with cutoffs for males at 8+ (hazardous drinking) and 15+ points (alcohol abuse). The full scales present a good internal consistency (α = .82) (Chen et al., 2024). In the present study, AUDIT had adequate reliability (ω = .72, α = .88).

Drug abuse was measured with the Drug Use Disorder Identification Test (DUDIT) (Hildebrand, 2015), a 5-point Likert scale including 11 items. The score ranges from 0 to 44 points, with a cutoff for males at 6+ points. Hildebrand (2015) reported a general excellent internal consistency in a variety of samples (α > .9). In the present study, DUDIT had adequate reliability (ω = .75, α = .93).

Depression was measured with Montgomery-Asberg Depression Rating Scale Self-report (MADRS-S; Bondolfi et al., 2010), a 7-point Likert scale with nine items. The scale ranges from 0 to 54 points with cutoffs at 13 (mild depression); 20 (moderate depression); and 35 (severe depression). The scale shows good to excellent internal consistency (α = .85–.94) (Bondolfi et al., 2010). In the present study, MADRS-S had adequate reliability (ω = .77, α = .88).

Multiple psychiatric conditions were screened for during the assessment interview using the Mini International Neuropsychiatric Interview (M.I.N.I.) 6.0.0 (Sheehan et al., 1998). The M.I.N.I. interview is based on the DSM-IV diagnostic criteria and includes anti-sociality, depression, generalized anxiety disorder, social anxiety, mania, hypomania, panic disorder, post-traumatic stress disorder (PTSD), psychosis, suicidality, obsessive-compulsive disorder (OCD), anorexia, bulimia, and binge eating.

The descriptive data, including the predictors in the regression models, originate from pre-treatment measurements in the Lätth et al. (2022) clinical trial. For the study outcomes, data from all trial measurement points are included: pre-treatment, seven weekly measures during the eight-week trial, post-treatment, and one-month follow-up.

### Procedure

We collected data during the assessment interview and through self-administered online questionnaires. Research team members (i.e. psychologists and a medical student) with training to perform the M.I.N.I. interview and access to consultation with a psychiatrist performed a 1- to 2-h-long assessment interview via chat before including a participant. After inclusion, participants gained access to the baseline measurement questionnaires through the study platform. In the same way, weekly-, post-, and follow-up measurements were collected through questionnaires over the treatment platform. To ensure the anonymity of participants, the platform was fully accessible via the Darknet, and all the main functionalities of the platform also worked well without enabling JavaScript. No identifiable information was collected, and no IP addresses were tracked. We offered no incentives to participants.

### Statistical Analysis

In a series of linear mixed effects models, we examined the extent to which CSAM viewing time, COPINE severity, and the age of the youngest child in the viewed material were associated with a set of sexuality-based measures. Specifically, we examined self-reported pedophilic interest, sexual sadistic interest, zoophilic interest, and hypersexuality traits. Several other paraphilic interests are potentially statistically related to the outcomes of interest, but to avoid model overfitting, we focused on those we regarded as most theoretically relevant. To account for the repeated measures of each outcome across time, the models included random intercepts for each participant. These models were fitted using the *lme4* package (Bates et al., 2015) for R (R Core Team, 2023), and Satterthwaite degrees of freedom were estimated using the *lmerTest* package (Kuznetsova et al., 2017). All models were estimated using restricted maximum likelihood. Missing observations were not imputed and were omitted from the analyses. Analyses used a significance threshold of .05. The sample size was determined based on a power analysis for the primary analysis of the trial, but we provide a sensitivity power analysis to contextualize what effects the present study is capable of detecting. The sample size for the present study provides 80% power to detect correlations of *r* = .22. This estimate assumes one observation per participant. For analyses involving repeated measures, the power will be greater, but it will vary from analysis to analysis depending on the number of observations available. Effect sizes were interpreted as small (*r* = .1–.3), medium (*r* = .3–.5), and large (*r* = .5–1.0). The authors take responsibility for the integrity of the data and have made every effort to avoid inflating statistically significant results.

## Results

**Figure 1.**
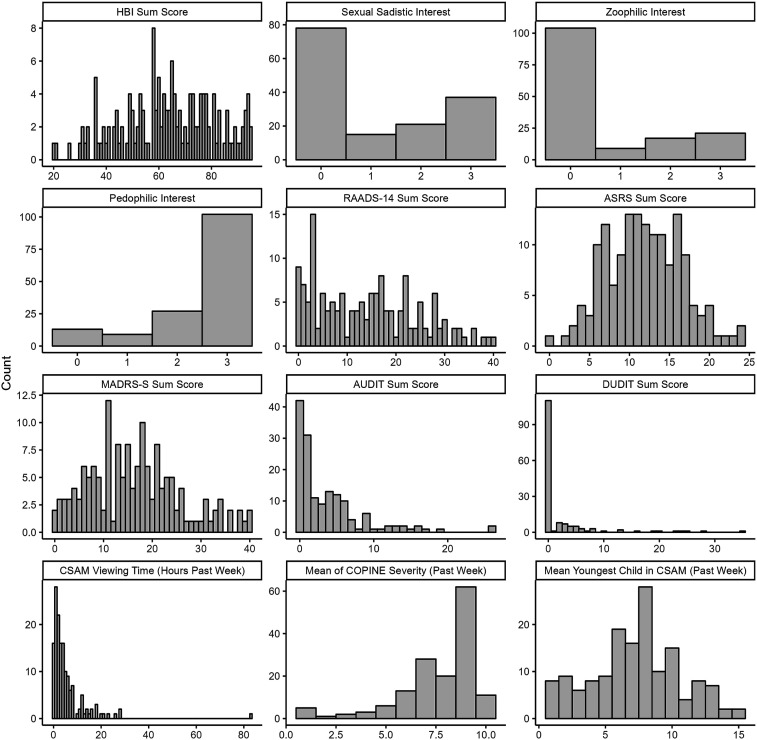
Distribution of Measured Variables. Note: Distributions of selected psychiatric variables, including predictors and outcomes. HBI-19, RAADS-14, ASRS, MADRS-S, AUDIT, and DUDIT are displayed as total scores. Response options for paraphilic interests are 0=completely disagree; 1=somewhat disagree; 2=somewhat agree; 3=completely agree, to having each interest. CSAM consumption is displayed as hours of viewing time in the past week, COPINE shows the mean score of the most severe material used in the past week, and the age (years) of the youngest child viewed in CSAM in the past week.

Several psychiatric conditions were commonly reported. In addition to the previously published (Lätth et al., 2022) prevalence of pedophilic interest (85%) and probable pedophilic disorder (60%), 38% reported a sexual sadistic interest, with 8% having a probable ongoing sexual sadism disorder. Twenty-five percent reported having a zoophilic interest, however, none met the criteria for an ongoing probable zoophilic disorder. As can be seen in Figure 1, the distributions of the paraphilic interests were overall skewed, such that most participants did not indicate sadistic or zoophilic interests. Hypersexuality was indicated in 74% of participants. Neuropsychiatric conditions were common, with autism traits indicated among 50% of participants (including 21% being above the higher cutoff of RAADS-14) and ADHD among 37%. The proportion reporting ongoing depression ranged from 34% (M.I.N.I.) to 65% (MADRS-S), with 33% indicating some level of suicidality. The most common anxiety disorders were social anxiety (18%) and generalized anxiety disorder (16%). Ten percent of participants reported anti-sociality. See Table 1 and Figure 1 for full data and measures.

**Figure 2.**
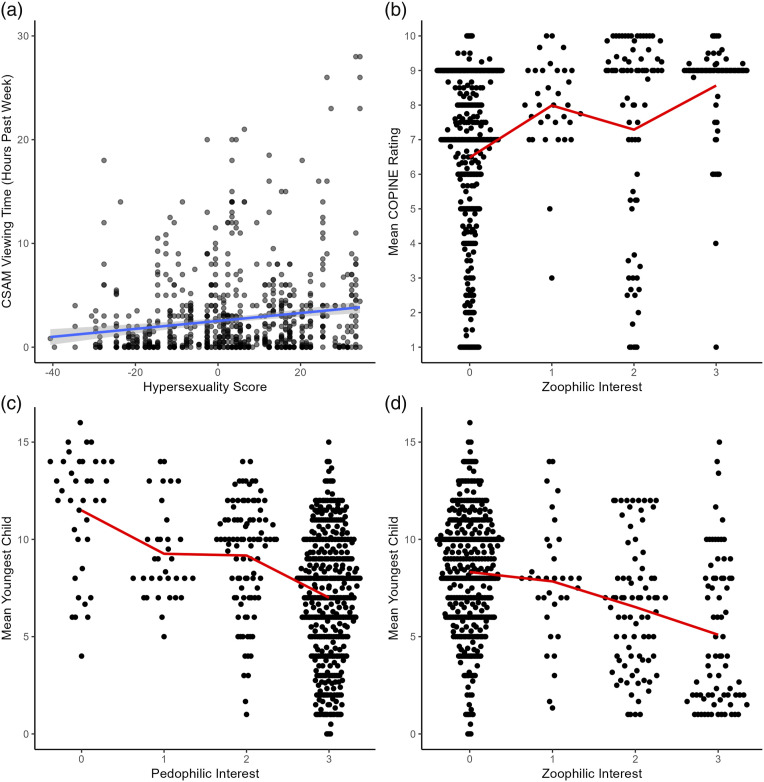
Sexuality-Based Predictors of Primary Outcomes.Note: Self-reported CSAM-related behaviors over the past seven days, predicted by hypersexuality, pedophilic interests, and zoophilic interests. (a) CSAM viewing time in the past week (hours) predicted by hypersexuality score (blue line represents linear regression predictions). (b) Severity of CSAM content (mean COPINE score of the most severe material used in the past week) predicted by sexual zoophilic interest (red line represents mean values, y-axis is truncated for legibility, COPINE scores are 1=Indicative, 2=Nudist, 3=Erotic, 4=Posing, 5=Erotic posing, 6=Explicit Erotic Posing, 7=Explicit Sexual Activity, 8=Assault, 9=Gross Assault,10=Sadistic/Bestiality). (c) Estimated age (years) of the youngest child in material predicted by pedophilic interest (red line represents mean values). (d) Estimated age (years) of the youngest child viewed the past week predicted by zoophilic interest (red line represents mean values).

The results of the mixed effects models examining the outcomes are presented in Table 2. Hypersexuality was associated with spending more time viewing CSAM (*r* = .295); zoophilic sexual interest was associated with viewing more severe CSAM (*r* = .195); zoophilic (*r* = −.218) and pedophilic (*r* = −.273) sexual interest were associated with viewing CSAM that included younger children. As shown, the effect sizes were overall small, but close to medium for the link between hypersexuality and CSAM time viewing, as well as the link between pedophilic interest and the age of children viewed in CSAM. These results are visually displayed in Figure 2. Additionally, sexual sadistic interest had a significant bivariate relationship with COPINE severity (*b* = 0.28, *SE* = 0.13, *t* (154.17) = 2.10, *p* = .04), but this coefficient became nonsignificant with the inclusion of zoophilic interest in the model. Because the COPINE severity scale explicitly notes bestiality at its highest value (i.e., 10), we assessed whether removing the observations *(n* = 29) with a value of 10 would change the relationship between zoophilic interests and COPINE severity. Removing these cases did not meaningfully change this relationship (*b* = 0.45, *SE* = 0.16, *t* (147.72) = 2.83, *p* = .005). We also tested models with interactions between each of the predictors. None of these models outperformed the models without interactions according to model fit indices and likelihood ratio tests, and there were no significant interactions.

**Table 2.** Linear Mixed Effects Models Predicting Primary Outcomes From Sexuality-Based Measures.

CSAM viewing time					
Predictor	*b*	*se*	*df*	*t*	*p*
Intercept	3.37	0.39	135.69	8.74	<.001
Pedophilic interest	0.71	0.42	136.87	1.71	.09
Sadistic sexual interest	−0.06	0.32	137.61	−0.18	.85
Zoophilic interest	−0.27	0.36	136.67	−0.74	.46
Hypersexuality	0.07	0.02	146.42	3.30	.001
Random effects	Variance				
Participant intercepts	18.77				
Residual	14.27				

COPINE severity					
Predictor	*b*	*se*	*df*	*t*	*p*
Intercept	7.25	0.17	150.18	43.32	<.001
Pedophilic interest	−0.09	0.18	160.35	−0.50	.62
Sadistic sexual interest	0.20	0.14	149.04	1.42	.16
Zoophilic interest	0.46	0.16	151.45	2.95	.003
Hypersexuality	−0.01	0.01	158.17	−1.05	.29
Random effects	Variance				
Participant intercepts	3.46				
Residual	1.98				

Age of youngest child					
Predictor	*b*	*se*	*df*	*t*	*p*
Intercept	7.70	0.24	141.59	32.64	<.001
Pedophilic interest	−1.01	0.26	148.08	−3.92	<.001
Sadistic sexual interest	−0.02	0.20	141.05	−0.11	.91
Zoophilic interest	−0.56	0.22	142.54	−2.51	.01
Hypersexuality	−0.01	0.01	147.00	−0.90	.37
Random effects	Variance				
Participant intercepts	7.35				
Residual	2.56				

*Note.* Predictors are mean-centred. Degrees of freedom are estimated using the Satterthwaite method. All models are estimated with REML.

## Discussion

Our first aim was to describe psychiatric morbidities among primarily male, mainly non-convicted and help-seeking CSAM users. We found that several morbidities were commonly reported. At least one-third reported some level of depression (the exact proportion varied between instruments), and a similar proportion reported some level of suicidality. Half screened positive for autism traits, and more than one-third for ADHD, strengthening the previously reported link between sexual interest in children and neurodevelopmental disorders (Abé et al., 2021). Hypersexuality was indicated for the majority of participants (74%). In addition to the known high prevalence of pedophilic interest (85%), a substantial proportion reported sexual sadistic (38%) or zoophilic interests (25%). In summary, this group of CSAM users presents not only sexual pathologies but also a broad range of other mental health needs within affective and neuropsychiatric domains.

Our second aim was to explore associations between paraphilic interests and hypersexuality, and specific aspects of the CSAM offense behaviors. We observed several links. Consistent with previous research on paraphilias and concordant behaviors (Seto et al., 2021), participants’ pedophilic interests were reflected in their CSAM use, here measured by the age of the youngest child depicted in CSAM. Similarly, individuals with higher hypersexuality scores spent more time engaging in CSAM use. Notably, zoophilic interest was associated with two aspects of CSAM content: more severe content and depictions of younger children. The associations were generally small to medium in magnitude, but given the importance of the outcomes we are examining, they are still potentially meaningful and have clinical relevance. The results imply that atypical sexuality is not just a motivational factor for child sexual offending generally (Seto, 2019) and a risk factor for sexual recidivism (Hanson & Morton-Bourgon, 2005), but could also be linked to specific aspects of CSAM-related criminal behavior. As with other CSA crimes (Seto, 2019), understanding CSAM use requires consideration of multiple factors beyond just pedophilia.

Among our findings, the associations between zoophilia and CSAM content are particularly noteworthy. This association remained even after excluding the most extreme COPINE scale item, which includes bestiality. Also, the association between CSAM severity and sexual sadistic interest became non-significant when including zoophilia in the same model. These results suggest that the presence of zoophilic interests indicates a risk not only for sexual offenses involving animals, but also for abusive behaviors in the more extreme end of the spectrum towards young children. The relevance of this is underpinned by a study by Garant and Proulx (2024), where about half of an online sample of people with pedophilic interests also reported some level of zoophilic fantasies. Interestingly, Zidenberg et al. (2025) recently reported a positive association between zoophilic fantasies and attitudes supporting rape in a community sample. Altogether, this highlights the importance of exploring how zoophilic interest may be linked to the risk of other sexual offending behaviors, beyond those involving animals.

Our results carry clinical implications. They suggest specialized clinics for individuals at risk for CSA-related offenses should be equipped to diagnose and treat not only the sexual pathologies traditionally associated with this group, such as pedophilia and hypersexuality, but also other paraphilias, with special attention to zoophilic interests. Further, other mental health needs such as depression, suicidality and neuropsychiatric conditions should be screened for. This may necessitate expanding intake assessments that traditionally address risk factors for sexual violence to a broader spectrum of mental health conditions and expanding the range of treatment options. Firstly, treatment delivery may need tailoring at an individual level (Bonta & Andrews, 2023), including adjusting the methods to mental health needs, such as incorporating breaks for patients with attention deficits and avoiding metaphorical language with patients exhibiting autistic traits. Secondly, these mental health needs can be important treatment targets in their own right. Addressing this broader range of conditions may provide value both for effectively achieving risk-reduction treatment goals and for meeting humanitarian objectives to offer treatment of psychiatric disorders to these individuals.

The associations between non-sexual psychiatric morbidities and sexual offending behavior should be further explored (Allely & Creaby-Attwood, 2016; Langevin et al., 2023). Interestingly, ADHD, as well as observed mood disorders, have previously been proposed to serve as disinhibitory factors for sexual behaviors (Kafka, 2012). While most individuals with neuropsychiatric disorders do not engage in sexual crimes, some of the diagnostic criteria seem to relate to established risk factors for sexual violence. Future research should explore whether specific autism-related traits, particularly social functioning, contribute to the well-established risk factor of intimacy deficits (Hanson & Morton-Bourgon, 2005). Additionally, it would be valuable to investigate whether impulsivity, a known risk factor for sexual violence, also serves as a risk factor for CSAM use when present in individuals with ADHD (Holcomb et al., 2019). The potential contribution of psychiatric knowledge to the forensic field warrants further investigation.

The psychiatric morbidities detected here also suggest that CSAM users presumably present to other mental health care services for symptoms seemingly unrelated to their sexual issues or offending behaviors. In this specific sample, more than half reported prior professional help for psychiatric conditions (Lätth et al., 2022). As such, the healthcare system may have an underused but potentially important role to play in detecting at-risk individuals for sexually offending within the broader psychiatric population. However, identifying these individuals requires multiple strategies, given that pedophilic interests are strongly connected to shame and stigma (Jahnke, 2018). One potential way forward would be to introduce screening for hypersexuality and paraphilias in selected populations seeking help for other mental health needs and to ensure access to specialized care.

### Limitations

Some study limitations need to be addressed. The results rely on self-reported data on highly sensitive and stigmatized topics. To reduce responding bias, thorough measures were taken to ensure anonymity, which we believe increased the likelihood of reporting stigmatized thoughts and behaviors, such as making the treatment platform available without leaving the encrypted Darknet browser Tor. To investigate data quality, we measured truthfulness in responding and found that participants reported high levels of truthfulness (Lätth et al., 2022). Further, some of the instruments were not validated for anonymous online use, and we did not have sufficient statistical power to investigate interaction effects. Another limitation regards the external generalizability of the results. Given that the participants were help-seeking and recruited mainly via the Darknet, their characteristics may differ from other CSAM users.

### Future Research

The findings presented in this article warrant replication in additional samples of CSAM users to confirm their validity. Future research should aim to further clarify the dynamics of CSAM offending by examining the roles of common psychiatric morbidities within this population, beyond atypical sexuality. This includes investigating the potential contributions of depression, anxiety, autism, and ADHD. In terms of treatment, it would be valuable to explore whether interventions targeting hypersexuality and other paraphilias, such as zoophilia, yield effects consistent with the study’s findings of reducing the frequency and severity of CSAM use.

### Conclusion

The participants of this study, a group of anonymous, primarily male, and mainly non-convicted CSAM users, mostly recruited over the Darknet, presented multiple psychiatric needs. Participants screened positive for several conditions, including paraphilias, hypersexuality, autism, ADHD, and depression. Furthermore, some of these morbidities were associated with specific aspects of the complex behavior of CSAM use. Hypersexuality was associated with CSAM viewing time, pedophilic interest was associated with the age of the youngest child in CSAM viewed, and zoophilia was associated with CSAM severity and the age of the youngest child viewed. Treatment could benefit from considering not only traditional sexual violence risk factors but also a broader range of mental health needs. Lastly, our findings suggest that the healthcare system holds the potential to improve its capacity to identify individuals at risk of engaging in sexual offending.

## Data Availability

Due to ethical restrictions, data are available only as part of a research collaboration approved by an ethics committee.*
